# Soluble gp130 inhibits Th17 polarization in neutrophilic asthma by blocking IL-6 *trans*-signaling in dendritic cells

**DOI:** 10.3389/fimmu.2026.1787115

**Published:** 2026-03-27

**Authors:** Suhua Zhu, Yun Yao, Tangfeng Lv, Feng Yao, Jing Xu, Ang Zhang, Fang Zhang

**Affiliations:** 1 Department of Respiratory and Critical Care Medicine, Jinling Hospital, Affiliated Hospital of Medical School, Nanjing University, Nanjing, China; 2 Department of Respiratory and Critical Care Medicine, Lujiang County People’s Hospital, Hefei, China; 3 Department of clinical pharmacology, the Second Affiliated Hospital of Anhui Medical University, Hefei, China

**Keywords:** dendritic cells, IL-23/IL-17 axis, IL-6 trans-signaling, neutrophilic asthma, soluble gp130 (sgp130), Th17polarization

## Abstract

Interleukin-6 (IL-6) *trans*-signaling modulates immune responses in asthma, yet the mechanisms linking this pathway to Th17 skewing in neutrophilic asthma remain incompletely defined. Here, we evaluated soluble gp130 (sgp130), a selective inhibitor of IL-6 *trans*-signaling, *in vivo* and *in vitro*. A murine neutrophilic asthma model was established by ovalbumin (OVA) sensitization followed by lipopolysaccharide (LPS) plus OVA challenge, and sgp130 was administered intratracheally. Airway neutrophilic inflammation, Th17/Treg responses, and IL-23 expression in lung dendritic cells (DCs) were assessed, and the contribution of Th17 cells was examined by adoptive transfer. In parallel, DCs were cocultured with naïve CD4^+^ T cells in the presence of Hyper-IL-6 (an IL-6/sIL-6R fusion protein that activates IL-6 *trans*-signaling) with or without sgp130 to quantify DC-derived IL-23 and Th17 differentiation; additionally, DCs conditioned with Hyper-IL-6 with or without sgp130 were delivered intratracheally to establish a DC-transfer asthma model. In neutrophilic asthma, bronchoalveolar lavage fluid (BALF) levels of IL-6/sIL-6R complex were elevated and positively associated with neutrophil counts and IL-17 production. Blockade of IL-6 *trans*-signaling with sgp130 attenuated airway neutrophilia, reduced Th17 polarization, increased Treg response, and decreased IL-23 expression in lung DCs, whereas adoptive transfer of Th17 cells partially abrogated these protective effects. Consistently, Hyper-IL-6 increased IL-23 expression in DCs and promoted Th17 differentiation *in vitro*, both of which were suppressed by sgp130. Moreover, airway transfer of Hyper-IL-6-conditioned DCs induced neutrophilic airway inflammation and Th17 polarization, while transfer of DCs conditioned with Hyper-IL-6 plus sgp130 markedly mitigated these responses. Collectively, IL-6 trans-signaling promotes Th17 polarization in neutrophilic asthma by enhancing DC IL-23 production, thereby driving neutrophilic airway inflammation, and selective inhibition with sgp130 may represent a mechanistically targeted therapeutic strategy.

## Introduction

Allergic asthma is a prevalent chronic inflammatory airway disorder characterized by allergen-triggered airflow obstruction and persistent airway inflammation (1). Despite substantial advances in defining its immunopathogenesis, clinical control remains suboptimal because of disease recurrence, frequent exacerbations, and corticosteroid resistance in certain patient subsets (2).This clinical heterogeneity is reflected in distinct inflammatory endotypes, most notably eosinophilic asthma dominated by T helper 2 (Th2) immunity and neutrophilic asthma in which T helper 17 (Th17) responses are prominent drivers of pathology (3, 4). Neutrophilic asthma is characterized by airway neutrophilia, enhanced Th17 polarization, and a high prevalence of corticosteroid resistance (5, 6). Although Th17 cells have been linked to corticosteroid resistance in neutrophilic asthma (7, 8), the upstream mechanisms that govern Th17 differentiation in this setting remain incompletely defined. Therefore, delineating the regulatory pathways that promote Th17 polarization in neutrophilic asthma is essential for identifying mechanism-based therapies to overcome corticosteroid resistance.

Interleukin-6 (IL-6) signaling is mediated through two principal pathways, classical signaling and trans-signaling, which differ in receptor usage and downstream biological effects (9, 10). In classical signaling, IL-6 binds the membrane-bound IL-6 receptor alpha (mIL-6Rα), leading to homodimerization of the ubiquitously expressed signal-transducing subunit gp130 and activation of downstream cascades, including JAK/STAT signaling (11, 12). By contrast, in *trans*-signaling, IL-6 associates with the soluble IL-6 receptor (sIL-6R) to form an IL-6/sIL-6R complex (also referred to as Hyper-IL-6), which activates gp130 on cells that do not express membrane IL-6R (13). These signaling modes are not functionally redundant and exert pleiotropic effects across physiological and pathological contexts (14). Accumulating evidence implicates IL-6 *trans*-signaling in inflammatory and autoimmune diseases, whereas classical signaling is more closely linked to homeostatic functions (15). In asthma pathogenesis, IL-6 signaling shapes inflammatory endotypes by differentially regulating Th2 and Th17 differentiation, with classical and *trans*-signaling mediating distinct biological effects (16). Asthma is commonly stratified into Th2-high disease, characterized by dominant Th2-driven responses, and Th2-low disease, which may involve Th17-associated inflammation. Classical IL-6 signaling has been shown to restrain Th2 differentiation in allergen-specific T cells by attenuating IL-2 signaling during early T cell activation, thereby limiting Th2 priming and downstream IgE-mediated allergic responses (17). By contrast, IL-6 *trans*-signaling is increasingly recognized as a key driver of pro-inflammatory programs in asthma, in large part because of its strong capacity to promote Th17 polarization. Mechanistically, IL-6/sIL-6R complex activates STAT3 phosphorylation and upregulate RORγt, the lineage-defining transcription factor for Th17 commitment, potentially within the context of the IL-6-STAT3 axis (18, 19). In experimental asthma models, IL-6 *trans*-signaling has been reported to exert dual pathogenic effects by enhancing airway epithelial activation and promoting Th2-low neutrophilic inflammation, a hallmark of severe steroid-refractory asthma (20, 21). Collectively, these observations support IL-6 *trans*-signaling as a therapeutically actionable pathway, particularly for neutrophilic asthma in which glucocorticoids often provide limited benefit.

Dendritic cells (DCs) are specialized antigen-presenting cells that critically shape T helper cell polarization in asthma by directing naïve T cells toward Th2 or Th17 fates (22, 23). In murine asthma models, DC function is highly context dependent and is strongly influenced by the inflammatory microenvironment; for example, damage-associated molecular patterns such as high-mobility group box 1 (HMGB1) can amplify DC-driven Th2/Th17 inflammation through STAT3-dependent pathways (24, 25), whereas IL-23 production by activated DCs preferentially supports Th17-associated pathology (26). Collectively, these findings position DCs as central regulators of asthma immunopathology by integrating environmental cues to differentially program Th2/Th17 responses and to modulate immune tolerance (27). In this study, we investigated whether sgp130 modulates IL-6 *trans*-signaling in DCs and thereby attenuates Th17 polarization in neutrophilic asthma. Our results support a pivotal role for IL-6 *trans*-signaling in neutrophilic asthma pathogenesis and indicate that sgp130 has therapeutic potential by selectively inhibiting this pathway.

## Materials and methods

### Animals and ethics

Female C57BL/6 mice (8–10 weeks old) were obtained from the Animal Center of Jinling Hospital and maintained under specific pathogen-free conditions. Mice were housed under standardized environmental parameters (20-22 °C, 50%-60% relative humidity, 12 hours light/dark cycle) with food and water provided ad libitum. All animal procedures were performed in accordance with the National Institutes of Health guidelines for the care and use of laboratory animals and were approved by the Institutional Animal Care and Use Committee of Nanjing University Medical School.

### Neutrophilic asthma model and intratracheal sgp130 administration

A murine model of neutrophilic asthma with Th17-skewed airway inflammation was established as previously described, with minor modifications (28). Female C57BL/6 mice (8–10 weeks old) were sensitized by intraperitoneal injection of ovalbumin (OVA; Grade V, Sigma-Aldrich, St. Louis, MO, USA; 20 µg) emulsified in aluminum hydroxide (alum; 2 mg) in 200 µL phosphate-buffered saline (PBS) on days 0 and 14. Mice were then challenged intratracheally with OVA (50 µg) on days 14-17; lipopolysaccharide (LPS; Escherichia coli serotype O26:B6, Sigma-Aldrich; 10 µg) was intratracheally co-administered on day 15 and 17 to augment neutrophilic inflammation. All analyses were performed 24 hours after the final challenge.

Mice were randomly allocated to four groups (n = 5 per group) using a computer-generated random number sequence: (i) Control group, PBS-sensitized and OVA-challenged; (ii) Asthma group, OVA-sensitized and OVA/LPS-challenged; (iii) MSA group, OVA-sensitized and OVA/LPS-challenged with intratracheal pretreatment using mouse serum albumin (MSA; Sigma-Aldrich; 200 µg/kg) 30 min before each OVA/LPS challenge on day 15 and 17; and (iv) sgp130 group, OVA-sensitized and OVA/LPS-challenged with intratracheal pretreatment using soluble gp130 (sgp130; R&D Systems; 200 µg/kg) 30 min before each OVA/LPS challenge on day 15 and 17. The intervention with sgp130 or the IgG control was administered during the established sensitization and challenge phase, timed to coincide with the peak of innate immune cell activation induced by OVA+LPS. The sgp130 dose was selected based on preliminary dose-ranging experiments (100-400 µg/kg) evaluating suppression of airway inflammation. Mice were sacrificed 24 hours after the final challenge, a timepoint that aligns with the expected peak of cytokine release (including the IL-6/sIL-6R complex), as validated in our preliminary experiments. Lungs from mice were harvested for downstream analyses, including histopathology. Histological scoring was performed by investigators blinded to group allocation.

### *In vitro* Th17 differentiation and adoptive transfer

Naïve CD4^+^ T cells were isolated from the spleens of mice using a Mouse CD4^+^ T Cell Isolation Kit (StemCell Technologies, Vancouver, Canada) according to the manufacturer’s instructions and seeded at 1 × 10^6^ cells per well in 24-well plates precoated with anti-CD3 (5µg/mL; R&D Systems) and anti-CD28 (2µg/mL; R&D Systems). Cells were cultured under Th17-polarizing conditions with IL-6 (20 ng/mL; PeproTech), IL-23 (10 ng/mL; BioLegend), TGF-β1 (5 ng/mL; R&D Systems), IFN-γ (10µg/mL; BioXCell) and IL-4 (10 µg/mL; BioXCell). Cultures were maintained at 37 °C in 5% CO_2_ for 3–5 days. Th17 differentiation was verified by flow cytometry, with IL-17A^+^ cells comprising >75% of the CD4^+^ population. For adoptive transfer experiments, the asthma model was established as previously described. *In vitro*-differentiated Th17 cells (5 × 10^6^) were delivered by intratracheal instillation on days 15 and 17 before OVA/LPS challenge. Mice were euthanized 24 hours after the final challenge, then BALF and lung tissues were collected for downstream analyses.

### BALF analysis

Differential cell counts and cytokine concentrations in BALF were assessed using standardized procedures. Briefly, lungs were lavaged three times with 0.75 mL of ice-cold, calcium- and magnesium-free Hank’s balanced salt solution supplemented with 0.1 mM EDTA. Pooled BALF samples were centrifuged at 300 × g for 5 min at 4 °C to separate cells from supernatants. The cell-free supernatants were aliquoted and stored at -80 °C for subsequent cytokine analysis. Cell pellets were resuspended in PBS and subjected to cytocentrifugation (Shandon Cytospin, Thermo Scientific, USA), followed by fixation and Diff-Quick staining (Kokusaishiyaku, Japan). Differential cell counts were determined by morphological evaluation of 300 cells per slide under light microscopy at 400× magnification. Concentrations of interleukin (IL)-23, IL-17A, IL-4, and interferon (IFN)-γ in BALF were quantified using commercially available enzyme-linked immunosorbent assay (ELISA) kits (eBioscience, USA) according to the manufacturer’s instructions.

### ELISA for detection of the IL-6/sIL-6R complex

The interleukin-6/soluble interleukin-6 receptor (IL-6/sIL-6R) complex was quantified using a sandwich ELISA with minor modifications as previously described (29). Briefly, 96-well microplates (Corning, NY, USA) were coated overnight at 4 °C with a monoclonal anti-IL-6 capture antibody (2 µg/mL in 0.1 M carbonate-bicarbonate buffer, pH 9.6; 100 µL per well). Plates were blocked with 1% bovine serum albumin in PBS for 2 hours at room temperature. After washing, samples or recombinant IL-6/sIL-6R complexes standards (R&D Systems) were added and incubated overnight at 4 °C. Bound complexes were detected using a guinea pig anti-sIL-6R polyclonal antibody, followed by an alkaline phosphatase-conjugated goat anti-guinea pig IgG secondary antibody (Sigma-Aldrich). After a final wash, p-nitrophenyl phosphate substrate (Sigma-Aldrich) was added, and absorbance was measured at 405 nm using a microplate reader (Bio-Rad, Hercules, CA, USA). Concentrations of IL-6/sIL-6R complex were determined by interpolation from a standard curve generated with recombinant standards.

### Histopathological analysis

For histopathological evaluation, non-lavaged lung lobes were fixed in 4% paraformaldehyde and paraffin-embedded according to standard protocols. Serial sections (5 µm) were prepared and stained with hematoxylin and eosin (H&E) to assess inflammatory cell infiltration and overall tissue architecture. Periodic acid-Schiff (PAS) staining was performed to identify and quantify mucus-producing goblet cells within the airway epithelium. PAS-positive cells were quantified in cross-sectional airway sections using light microscopy at 200× magnification. For each experimental group, lungs from five to six mice were analyzed, with six to eight randomly selected fields evaluated per section. All histological assessments were performed in a blinded manner.

### Culture and treatment of bone marrow-derived dendritic cells

BMDCs were generated according to established protocols with minor modifications (30). Briefly, bone marrow cells were aseptically harvested from the femurs and tibiae of 6–8-week-old C57BL/6 mice and cultured for 8 days in serum-free complete DC medium supplemented with recombinant murine granulocyte–macrophage colony-stimulating factor (GM-CSF; 20 ng/mL; R&D Systems) and interleukin-4 (IL-4; 10 ng/mL; R&D Systems). On day 8, CD11c^+^ cells were positively selected using anti-CD11c microbeads (Miltenyi Biotec, Auburn, CA, USA) by magnetic-activated cell sorting, yielding a purity of >95% as confirmed by flow cytometric analysis of CD11c expression. Purified BMDCs were rest for overnight, then stimulated for 48 hours under the following conditions: Hyper-IL-6 (100 nM; a recombinant fusion protein of IL-6 and soluble IL-6 receptor α; R&D Systems), Hyper-IL-6 in combination with sgp130 (200 or 400 ng/mL; R&D Systems). After stimulation, culture supernatants were collected, and interleukin-23 (IL-23) levels were quantified using a commercially available ELISA kit (R&D Systems) according to the manufacturer’s instructions. Hyper-IL-6 and sgp130 preparations were verified to be endotoxin-free (<0.1 endotoxin units/mL) using a Limulus amebocyte lysate assay (ZhanJiang A&C Biological, China). Cell viability was routinely assessed by trypan blue exclusion and consistently exceeded 95%.

### Coculture of dendritic cells with CD4^+^ T cells

A dendritic cell (DC)-CD4^+^ T cell coculture system was established to assess antigen-specific Th17 responses. CD4^+^ T cells were isolated from the spleens of ovalbumin (OVA)-sensitized mice by magnetic bead as previously described. Purified CD4^+^ T cells (1 × 10^5^ cells per well) were cocultured with BMDCs (2.5 × 10^4^ cells per well) in 96-well flat-bottom plates. Prior to coculture, BMDCs were prestimulated for 24 hours with Hyper-IL-6 (100 nM) in the presence or absence of sgp130 (200 or 400 ng/mL). Antigen-specific T cell activation was initiated by the addition of OVA (10µg/mL) to the coculture system. After 5 days of incubation at 37 °C in 5% CO_2_, culture supernatants were collected for IL-17A quantification using a commercial ELISA kit (R&D Systems). In parallel, cells were harvested for intracellular IL-17 staining followed by flow cytometric analysis to determine the frequency of IL-17-producing CD4^+^ T cells.

### Flow cytometric analysis

Single-cell suspensions from lung tissue were prepared as previously described (31) and used for intracellular cytokine staining to quantify IL-17-producing CD4^+^ T cells. Briefly, lung cells were adjusted to 4 × 10^6^ cells/mL and washed three times with FACS buffer (PBS supplemented with 1% bovine serum albumin and 0.1% sodium azide). Cells were treated with brefeldin A to inhibit cytokine secretion, followed by surface staining with LIVE/DEAD Fixable stains, anti-CD3-APC and anti-CD4-FITC antibodies (eBioscience, San Diego, CA, USA). After fixation and permeabilization, intracellular IL-17A or FoxP3 was detected using a PE-conjugated anti-IL-17 or FoxP3 monoclonal antibody (eBioscience) (see **Supplementary Figures S1**, **S2**). Data were acquired on a FACSCalibur flow cytometer (BD Biosciences), and CD3^+^CD4^+^IL-17^+^ Th17 cells or CD3^+^CD4^+^FoxP3^+^ Treg cells were quantified as a percentage of total CD3^+^ T cells.

To analysis the IL-6R and IL-23 expression in CD11c^+^ antigen-presenting cells (APCs), Low-density lung cells, enriched for mononuclear populations including APCs, were isolated via discontinuous Percoll gradient centrifugation as previously described (32). For surface marker analysis, Low-density cells were stained with an APC-conjugated anti-CD45 monoclonal antibody (mAb) (eBioscience), a FITC-conjugated anti-CD11c mAb (eBioscience), and a PE-conjugated anti-IL-6R mAb (Sigma-Aldrich, St. Louis, MO, USA) for 30 min at 4 °C. For intracellular IL-23 detection, cells were fixed and permeabilized using a commercial fixation/permeabilization kit (eBioscience) according to the manufacturer’s protocol, followed by staining with a PE-conjugated anti-IL-23 mAb (eBioscience) for 30 min at 4 °C (see **Supplementary Figures S3**, **S4**). All stained cells were analyzed using FACSCalibur flow cytometer (BD Biosciences).

For coculture experiments, cells from the DC-T cell coculture system were treated with brefeldin A (10 µg/mL; eBioscience) for 2 h, stained with a FITC-conjugated anti-CD4 monoclonal antibody (eBioscience) for 30 min at 4 °C, fixed and permeabilized using a commercial fixation/permeabilization buffer (eBioscience), and incubated with a PE-conjugated anti-IL-17 monoclonal antibody (eBioscience) for 30 min. Flow cytometric data were acquired using a FACSCalibur instrument (BD Biosciences) and analyzed using standard gating strategies to determine the frequency of IL-17-producing CD4^+^ T cells.

### Adoptive transfer of ovalbumin-loaded dendritic cells in a murine asthma model

An ovalbumin (OVA)-induced allergic asthma model was established in C57BL/6 mice by adoptive transfer of BMDCs as previously described (33). CD11c^+^ BMDCs were isolated by magnetic beads sorting and pulsed overnight with OVA (100 µg/mL; Grade V, Sigma-Aldrich) to generate antigen-loaded DCs. Subsequently, 2 × 10^6^ OVA-pulsed DCs suspended in 50 µL PBS were administered by intratracheal instillation under isoflurane anesthesia. Ten days after DC transfer, mice were challenged with aerosolized OVA [1% (w/v)] for 30 min daily for three consecutive days using an ultrasonic nebulizer. Animals were randomly assigned to three groups (n = 5 per group): (i) PBS/DC control group, receiving DCs pulsed with PBS alone; (ii) Hyper-IL-6/OVA-DCs group, receiving OVA-pulsed DCs pretreated with Hyper-IL-6 (100 nM); and (iii) Hyper-IL-6 + sgp130/OVA-DCs group, receiving OVA-pulsed DCs pretreated with Hyper-IL-6 (100 nM) in combination with sgp130 (400 ng/mL). Mice were euthanized 24 hours after the final challenge by CO_2_ asphyxiation, then BALF and lung tissues were collected for subsequent analyses.

### Statistical analysis

All quantitative data are presented as the mean ± standard error of the mean (SEM). Statistical analyses were performed using IBM SPSS Statistics software (version 23). Comparisons between two groups were conducted using an unpaired two-tailed Student’s t test. For comparisons involving multiple groups, one-way analysis of variance (ANOVA) was applied, followed by Dunnett’s *post hoc* test for multiple comparisons. A p value < 0.05 was considered statistically significant.

## Results

### Elevated IL-6/sIL-6R complex levels in BALF from asthmatic mice

**Figure 1:**
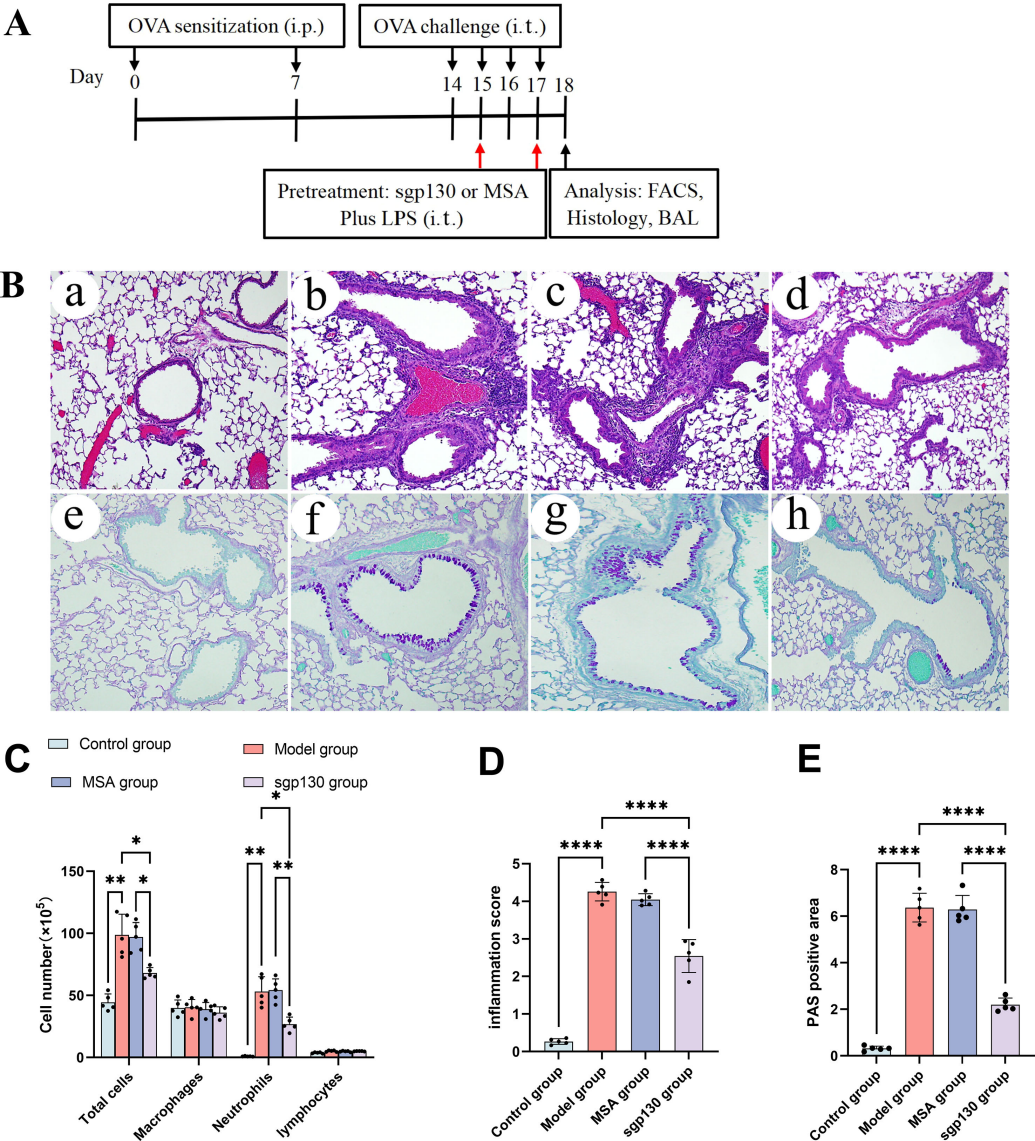
Local intratracheal administration of sgp130 attenuates neutrophilic airway inflammation and mucus hypersecretion in a murine asthma model. **(A)** Schematic overview of the experimental protocol for the administration of sgp130 in the murine model of neutrophilic asthma. **(B)** Representative histopathological analysis of lung tissue sections. Hematoxylin and eosin (H&E) staining (upper panels) shows marked peribronchial and perivascular inflammatory cell infiltration in OVA/LPS-challenged mice, which is attenuated following sgp130 treatment. Periodic acid-Schiff (PAS) staining (lower panels) demonstrates reduced goblet cell hyperplasia and mucus production in sgp130-treated mice compared with vehicle-treated controls. Representative images are shown for (a, e) control, (b, f) asthma, (c, g) MSA-treated, and (d, h) sgp130-treated groups. **(C)** Differential cell counts in BALF collected 24 h after the final OVA challenge. sgp130 treatment significantly reduced total inflammatory cell numbers, with a pronounced decrease in total cells and neutrophils, compared with vehicle-treated mice. Data are presented as mean ± SEM (n = 5 per group); *P < 0.05, **P < 0.01 versus the asthma group. **(D)** Semiquantitative assessment of peribronchial inflammation (scale 0-4) reveals a significant reduction in sgp130-treated mice. Data are presented as mean ± SEM (n = 5); ****P < 0.0001, versus the asthma group. **(E)** Quantification of PAS-positive mucus-producing areas expressed as a percentage of the total airway epithelial area, demonstrating significant suppression of airway mucus hypersecretion following sgp130 treatment. Data are presented as mean ± SEM (n = 5); ****P < 0.0001 versus the asthma group.

**Figure 2:**
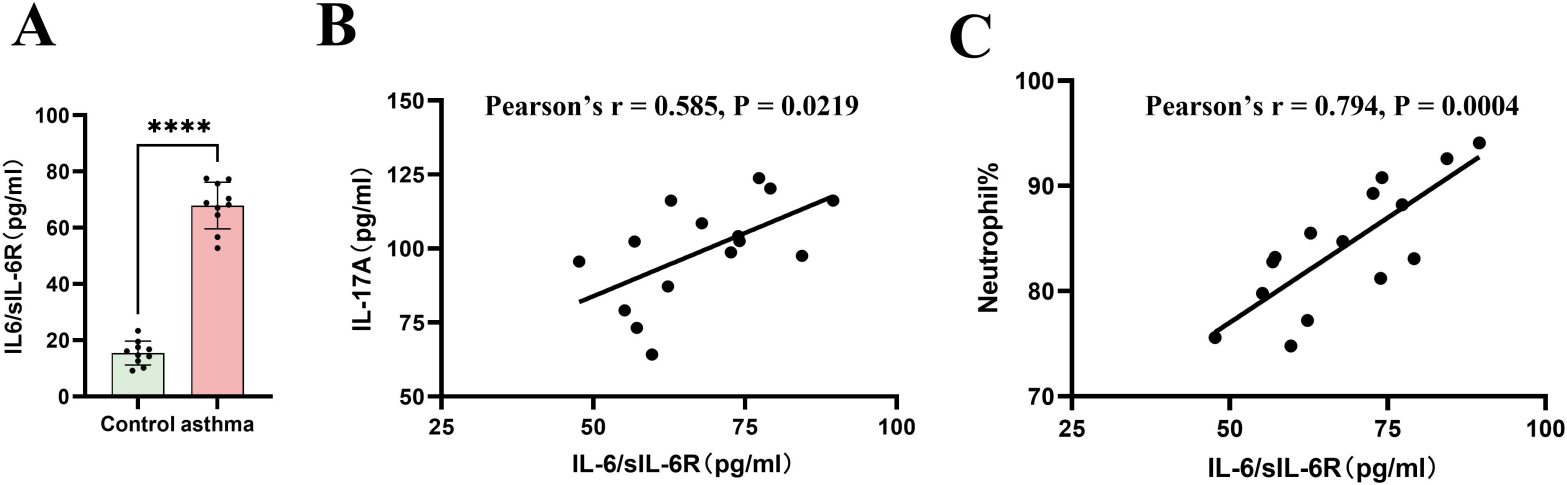
Elevated IL-6/sIL-6R complex levels correlate with neutrophilic inflammation in a murine model of neutrophilic asthma. **(A)** Concentrations of the IL-6/sIL-6R complex in BALF were quantified by ELISA and were significantly increased in OVA/LPS-challenged mice compared with PBS/OVA controls (****P < 0.0001; n = 10). Data are presented as mean ± SEM. **(B)** Correlation analysis showing a positive association between BALF IL-6/sIL-6R complex levels and IL-17 concentrations (Pearson’s r = 0.585, P < 0.05). **(C)** Correlation between BALF IL-6/sIL-6R complex levels and neutrophil percent in BALF (Pearson’s r = 0.794, P < 0.001) in OVA/LPS-challenged mice (n = 15).

To investigate the involvement of IL-6 trans-signaling in Th17-associated airway inflammation, a murine model of neutrophilic asthma was established by ovalbumin (OVA) sensitization with aluminum hydroxide (alum), followed by combined OVA and lipopolysaccharide (LPS) airway challenges (**Figure 1A**). Quantitative ELISA analysis demonstrated that levels of the IL-6/sIL-6R complex in BALF were significantly increased in OVA/LPS-challenged mice compared with PBS/OVA control mice (**Figure 2A**).

Notably, elevated BALF IL-6/sIL-6R complex levels were positively correlated with airway neutrophil counts (**Figure 2B**) and IL-17 concentrations (**Figure 2C**). These results indicate that enhanced IL-6 *trans*-signaling activity is closely associated with Th17-skewed immune responses and neutrophilic airway inflammation in this experimental asthma model.

### Local administration of sgp130 attenuates neutrophilic airway inflammation in a murine asthma model

Given the elevated IL-6/sIL-6R complex levels observed in asthmatic mice and their association with airway neutrophilia and IL-17 production, we next examined whether blockade of IL-6 *trans*-signaling by sgp130 could attenuate neutrophilic airway inflammation. To this end, sgp130 or mouse serum albumin (MSA) was administered locally by intratracheal instillation prior to OVA/LPS challenge.

Histopathological examination further supported the anti-inflammatory effects of sgp130. Hematoxylin and eosin (H&E) staining revealed pronounced peribronchial and perivascular inflammatory cell infiltration in OVA/LPS-challenged mice, whereas these pathological features were markedly attenuated in sgp130-treated animals (**Figures 1B, D**). In addition, periodic acid-Schiff (PAS) staining demonstrated a significant reduction in goblet cell hyperplasia and mucus production following sgp130 administration compared with MSA-treated controls (**Figures 1B, E**). As expected, OVA/LPS-challenged mice displayed a marked increase in total BALF cellularity, characterized predominantly by neutrophils with minimal eosinophil infiltration, consistent with a neutrophilic asthma phenotype. Intratracheal pretreatment with sgp130 significantly reduced total BALF cell counts and neutrophil numbers compared with MSA-treated controls (**Figure 1C**). Collectively, these results indicate that local intratracheal delivery of sgp130 effectively alleviates key pathological features of neutrophilic airway inflammation in this experimental asthma model.

### Immunomodulatory effects of sgp130 on Th17/Treg imbalance responses *in vivo*

**Figure 3:**
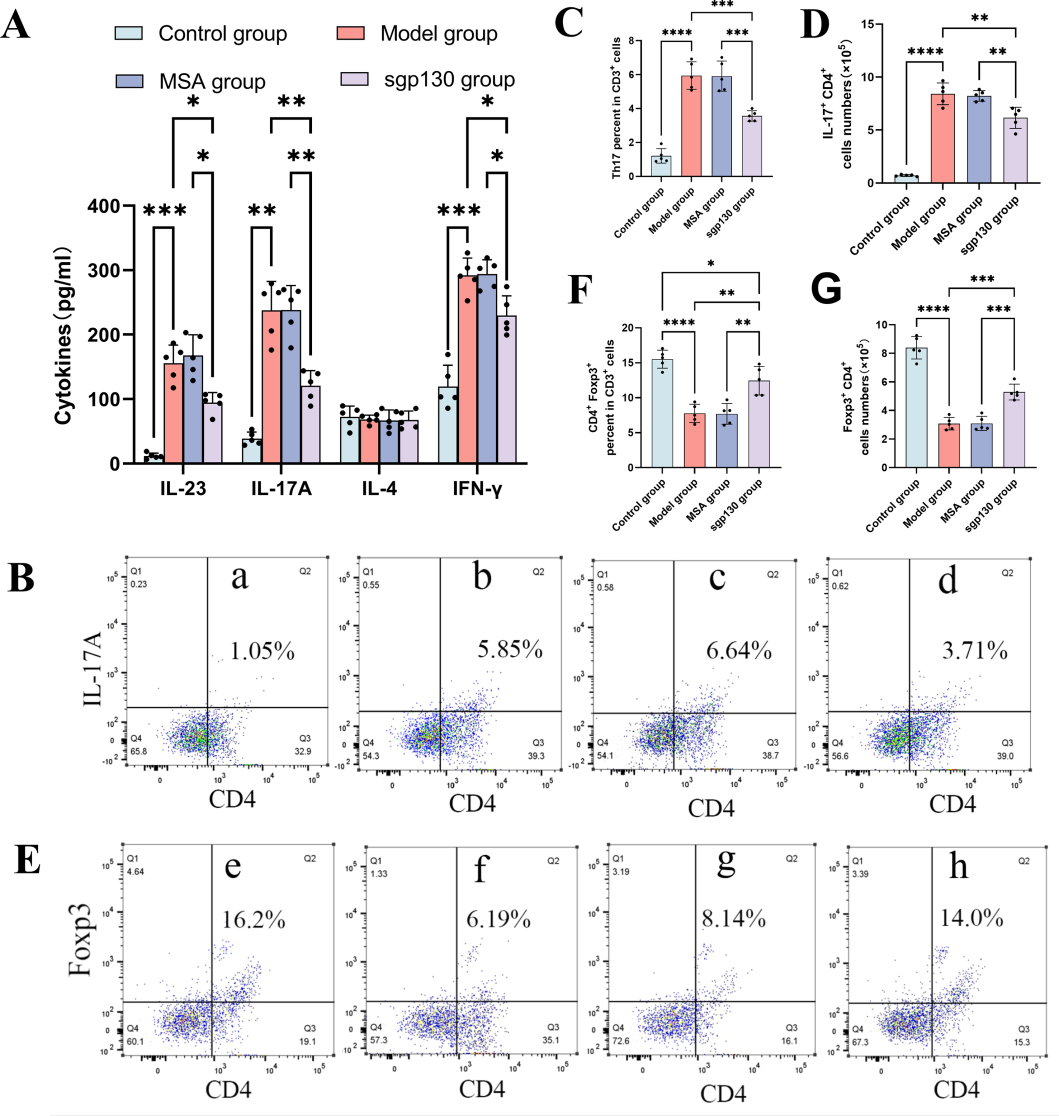
Intratracheal administration of sgp130 alleviates pulmonary Th17/Treg imbalance responses in a murine asthma model. **(A)** Cytokine concentrations in BALF collected 24 h after the final OVA challenge. ELISA analysis shows significant reductions in IL-23, IL-17A, and IFN-γ levels following sgp130 treatment, whereas IL-4 levels remain unchanged. Data are presented as mean ± SEM (n = 5); *P < 0.05, **P < 0.01 versus the asthma group. **(B)** Representative flow cytometric plots showing Th17 cells (CD3^+^CD4^+^IL-17A^+^) in lung single-cell suspensions from (a) control, (b) asthma, (c) MSA-treated, and (d) sgp130-treated mice. **(C)** Quantitative analysis of pulmonary Th17 percentage in total CD3^+^ T cells demonstrating a significant reduction following sgp130 treatment. Data are presented as mean ± SEM (n = 5); ***P < 0.001, ****P < 0.0001, versus the asthma group; **(D)** Quantitative analysis of absolute number of pulmonary Th17 cells demonstrating a significant reduction following sgp130 treatment. Data are presented as mean ± SEM (n = 5); **P < 0.01, ****P < 0.0001 versus the asthma group. **(E)** Representative flow cytometric plots showing CD3^+^CD4^+^Foxp3^+^ Treg populations in lung single-cell suspensions from (e) control, (f) asthma, (g) MSA-treated, and (h) sgp130-treated mice. **(F)** Quantitative analysis of the percentage of CD4^+^Foxp3^+^ Treg percentage in total CD3^+^ T cells demonstrating a significant increase following sgp130 treatment. Data are presented as mean ± SEM (n = 5); **P < 0.01, ****P < 0.0001 versus the asthma group; **(G)** Quantitative analysis of absolute number of pulmonary CD3^+^CD4^+^Foxp3^+^ Treg demonstrating a significant increase following sgp130 treatment. Data are presented as mean ± SEM (n = 5); ***P < 0.001, ****P < 0.0001 versus the asthma group.

To further characterize airway immune responses, cytokine profiles in BALF were quantitatively assessed. Intratracheal administration of sgp130 resulted in a significant reduction in Th17-associated cytokines, including IL-17A and IL-23, as well as the Th1-associated cytokine IFN-γ, compared with vehicle-treated asthmatic mice (**Figure 3A**). In contrast, levels of the Th2-associated cytokine IL-4 were not significantly altered, indicating a selective effect of sgp130 on Th17- and Th1-skewed inflammatory responses. Given the marked decrease in IL-17A levels in BALF following sgp130 treatment, we next examined its impact on pulmonary Th17 cell accumulation. Flow cytometric analysis demonstrated a pronounced increase the Th17 percentage in total CD3^+^ T cells (**Figures 3B, C**) and absolute Th17 cells number (**Figures 3B, D**) in lung tissue from OVA/LPS-challenged mice. Notably, sgp130 treatment significantly reduced the frequency and absolute number of pulmonary Th17 cells (**Figures 3B–D**). These findings indicate that local blockade of IL-6 *trans*-signaling by sgp130 effectively suppresses Th17 polarization *in vivo* in a murine model of neutrophilic asthma.

To further delineate the immunomodulatory effects of sgp130 in neutrophilic asthma, we assessed its impact on pulmonary regulatory T cell (Treg) responses. Flow cytometric analysis demonstrated that OVA/LPS challenge was associated with a reduction in Treg frequencies in lung tissue, whereas intratracheal administration of sgp130 significantly increased the proportion and absolute number of pulmonary Tregs (**Figures 3F, G**). Specifically, sgp130-treated mice exhibited a marked expansion of CD3^+^CD4^+^Foxp3^+^ T cells compared with vehicle-treated asthmatic controls (**Figures 3F, G**). These findings indicate that local inhibition of IL-6 *trans*-signaling by sgp130 is accompanied by enhanced Treg responses *in vivo*, suggesting a shift toward a more immunoregulatory airway microenvironment in this model of neutrophilic asthma.

### Adoptive transfer of Th17 cells abrogates the protective effects of sgp130 *in vivo*

**Figure 4:**
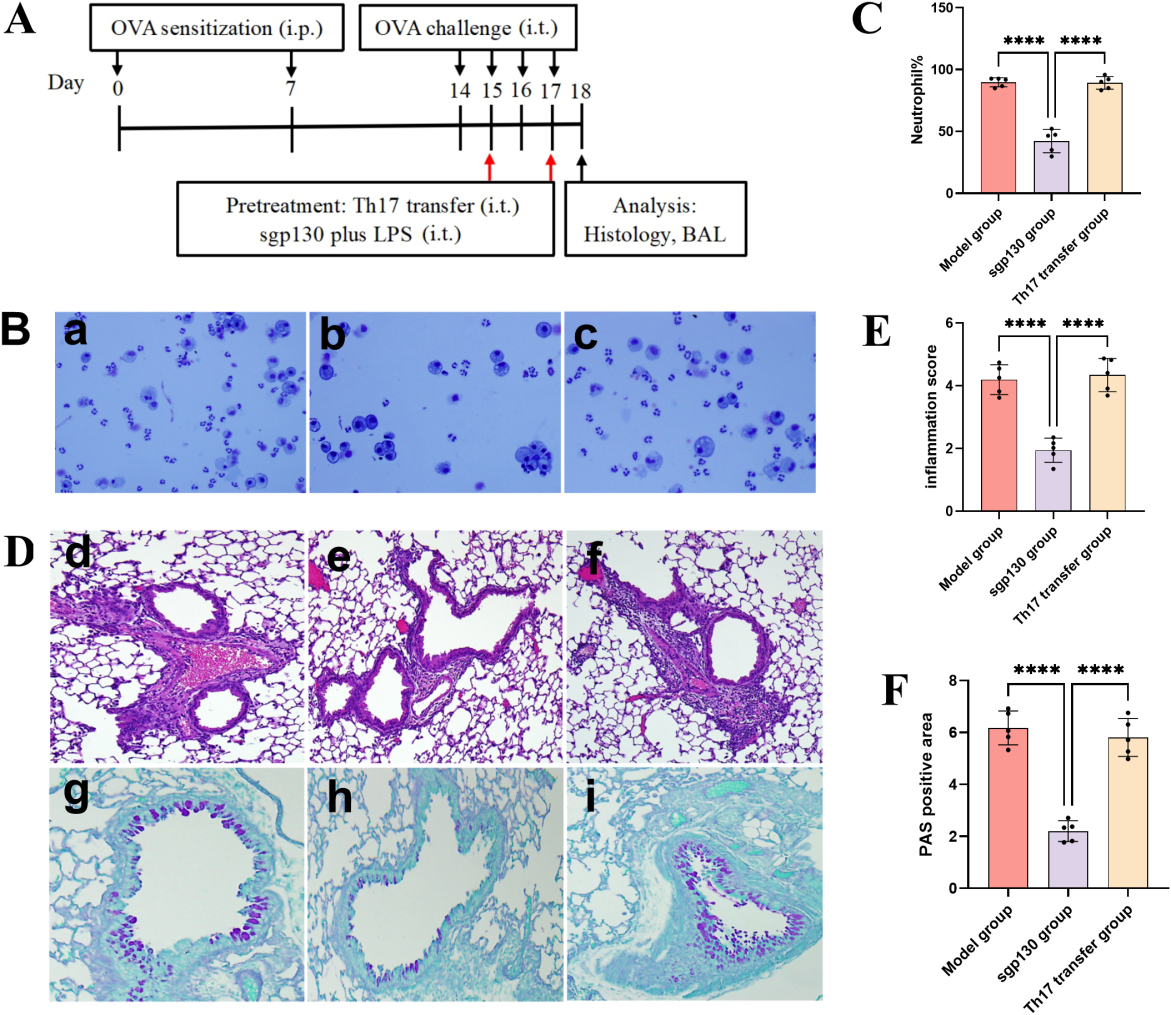
Intratracheal adoptive transfer of Th17 cells abrogates the protective effects of sgp130. **(A)** Schematic overview of the experimental protocol for adoptive transfer of Th17 cells in the murine model of neutrophilic asthma. **(B)** Representative Diff-Quick stained cytospin preparations of BALF cells from (a) asthma, (b) sgp130-treated, and (c) Th17-transferred mice. **(C)** Quantitative analysis of neutrophil percentages in BALF showing a marked increase following Th17 cell transfer. Data are presented as mean ± SEM (n = 5); ****P < 0.0001 versus the sgp130-treated group. **(D)** Representative histopathological analysis of lung tissues. Hematoxylin and eosin (H&E) staining (upper panels) illustrates increased inflammatory cell infiltration in Th17-transferred mice compared with sgp130-treated controls. Periodic acid-Schiff (PAS) staining (lower panels) shows enhanced mucus production following Th17 cell transfer. Images are shown for (d, g) asthma, (e, h) sgp130-treated, and (f, i) Th17-transferred groups. **(E)** Semiquantitative scoring of peribronchial inflammation (scale 0-4) demonstrating significant exacerbation in Th17-transferred mice. Data are presented as mean ± SEM (n = 5); ****P < 0.0001 versus the sgp130-treated group. **(F)** Quantification of PAS-positive mucus-producing areas expressed as a percentage of the airway epithelial area, showing Th17 transfer-mediated increases in airway mucus hypersecretion. Data are presented as mean ± SEM (n = 5); ****P < 0.0001 versus the sgp130-treated group.

To directly assess whether suppression of Th17 responses is required for the protective effects of sgp130, adoptive transfer experiments were performed in a murine model of neutrophilic asthma. *In vitro*-differentiated Th17 cells were intratracheally transferred into sgp130-treated mice prior to OVA/LPS challenge (**Figure 4A**). Adoptive transfer of Th17 cells markedly increased neutrophil accumulation in BALF compared with sgp130-treated mice without Th17 transfer (**Figures 4B, C**). Consistent with these findings, histopathological analysis revealed exacerbated airway inflammation in Th17-transferred mice, as evidenced by increased peribronchial and perivascular inflammatory cell infiltration (**Figures 4D, E**). In addition, periodic acid-Schiff (PAS) staining demonstrated enhanced goblet cell hyperplasia and mucus production following Th17 cell transfer, effectively reversing the attenuation of mucus hypersecretion observed with sgp130 treatment alone (**Figures 4D, F**). Collectively, these results demonstrate that adoptive transfer of Th17 cells counteracts the protective effects of sgp130 on neutrophilic airway inflammation, indicating that inhibition of Th17 responses is a critical mechanism underlying sgp130-mediated immunomodulation in neutrophilic asthma.

### sgp130 effect on IL-6R and IL-23 expression on pulmonary CD11c^+^ antigen-presenting cells

**Figure 5:**
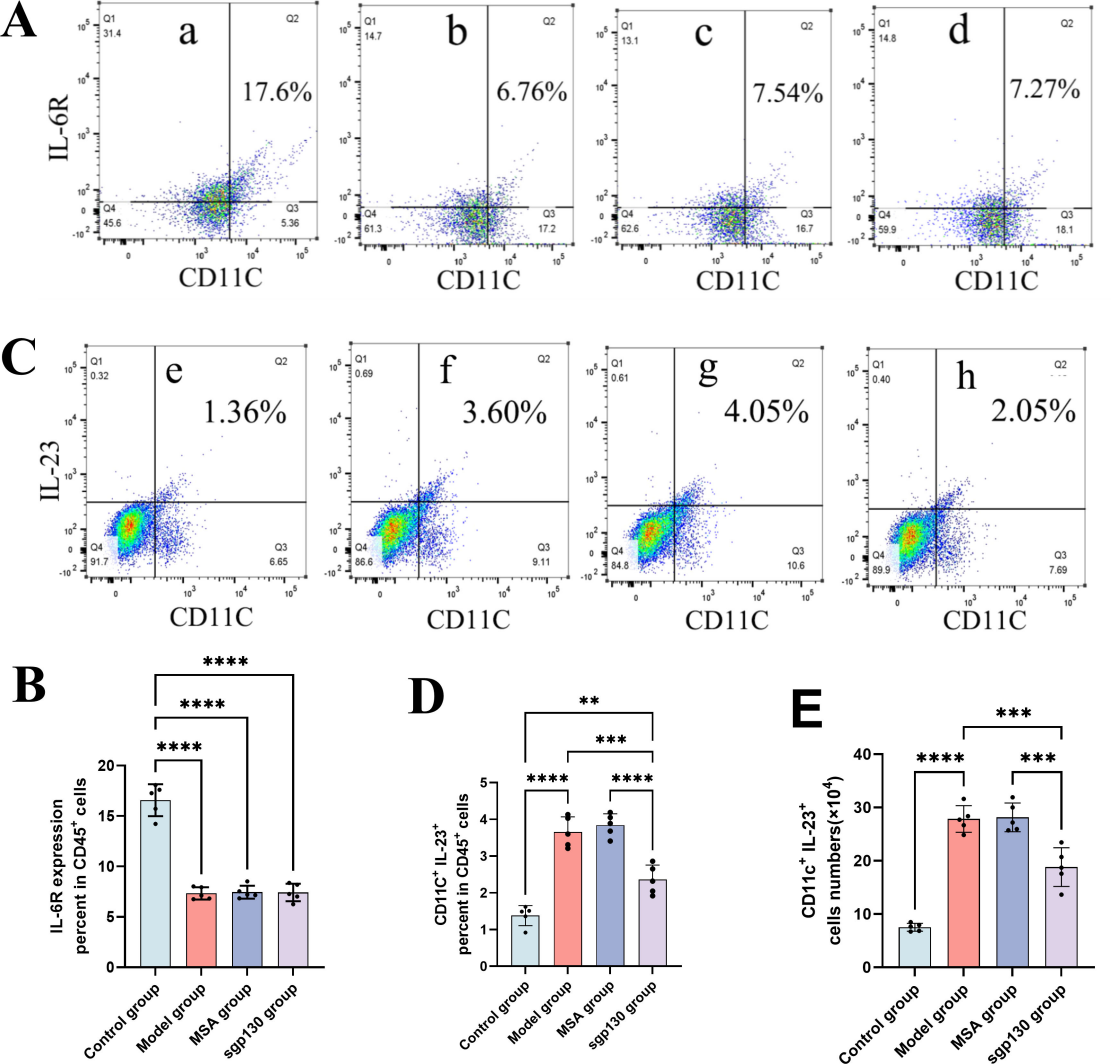
sgp130 effect on IL-6R and IL-23 expression on pulmonary CD11c^+^ antigen-presenting cells. **(A)** Representative flow cytometric plots showing IL-6R^+^ CD11c^+^ APC populations among CD45^+^ lung cells from (a) control, (b) asthma, (c) MSA-treated, and (d) sgp130-treated mice. **(B)** Quantitative analysis of the percentage of IL-6R^+^ CD11c^+^ APCs among CD45^+^ lung cells demonstrates a significant reduction in asthmatic mice, with no further change following sgp130 treatment. Data are presented as mean ± SEM (n = 5); ****P < 0.0001 versus the asthma group. **(C)** Representative flow cytometric plots showing IL-23^+^ CD11c^+^ APCs among CD45^+^ lung cells from (e) control, (f) asthma, (g) MSA-treated, and (h) sgp130-treated mice. **(D)** Quantitative analysis of the percentage of IL-23^+^ CD11c^+^ APCs among CD45^+^ lung cells demonstrates a significant reduction in sgp130-treated mice. Data are presented as mean ± SEM (n = 5); ***P < 0.001, ****P < 0.0001 versus the asthma group. **(E)** Quantitative analysis of the absolute number of IL-23^+^ CD11c^+^ APCs in lung tissue demonstrates a significant reduction following sgp130 treatment. Data are presented as mean ± SEM (n = 5); ***P < 0.001, ****P < 0.0001 versus the asthma group.

Because membrane-bound interleukin-6 receptor (IL-6R) expression is required for classical IL-6 signaling, we next examined IL-6R expression on pulmonary CD11c^+^ antigen-presenting cells (APCs) in the neutrophilic asthma model. Flow cytometric analysis revealed a significant reduction in the proportion of IL-6R^+^ CD11c^+^ APCs in lung tissue from OVA/LPS-challenged mice compared with control animals (**Figures 5A, B**). Notably, intratracheal administration of sgp130 did not significantly alter IL-6R expression on CD11c^+^ APCs relative to vehicle-treated asthmatic mice. These results indicate that neutrophilic airway inflammation is associated with reduced surface IL-6R expression on pulmonary APCs, while sgp130 exerts its immunomodulatory effects without directly modulating IL-6R expression levels on these cells.

Given the central role of dendritic cells (DCs) in T helper cell polarization, we next examined whether pulmonary CD11c^+^ APCs mediate the inhibitory effects of sgp130 on Th17 responses *in vivo*. Flow cytometric analysis revealed that OVA/LPS challenge markedly increased IL-23 expression in pulmonary CD11c^+^ APCs, whereas intratracheal administration of sgp130 significantly reduced both the frequency (**Figures 5C, D**) and absolute number (**Figure 5E**) of IL-23-producing CD11c^+^ APCs compared with vehicle-treated asthmatic mice. Consistent with the reduced IL-23 expression observed in pulmonary APCs, sgp130 treatment also resulted in a significant decrease in IL-23 concentrations in BALF (**Figure 3A**). These findings indicate that local inhibition of IL-6 *trans*-signaling by sgp130 is associated with suppressed IL-23 production by lung APCs, a cytokine axis that is critical for the differentiation and expansion of Th17 cells. Collectively, these data identify pulmonary CD11c^+^ APCs as a key cellular target through which sgp130 attenuates Th17-mediated immune responses in neutrophilic asthma.

### sgp130 effectively suppresses IL-6 *trans*-signaling-mediated Th17 polarization induced by Hyper-IL-6-activated BMDCs *in vitro*

**Figure 6:**
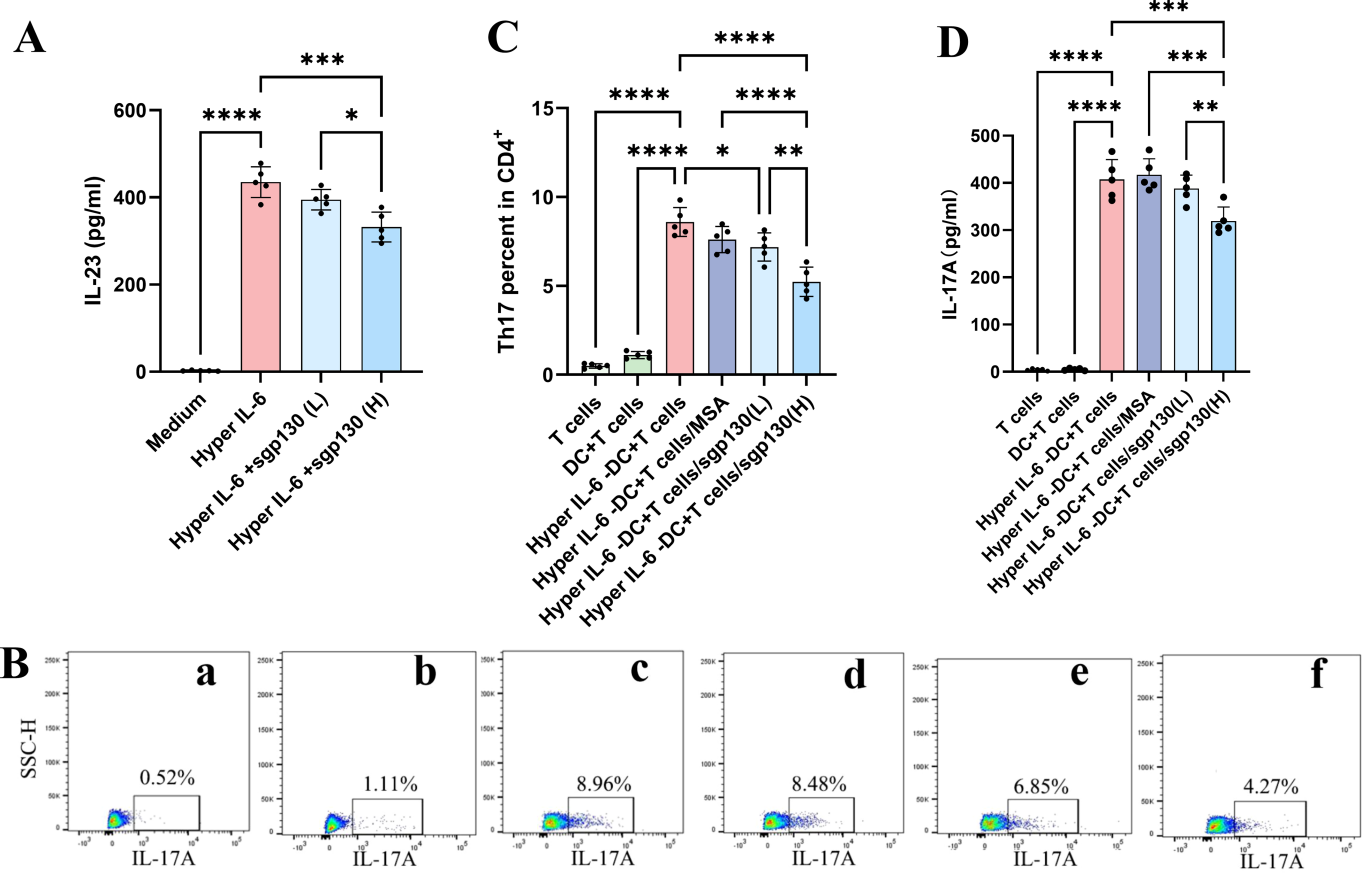
sgp130 attenuates Hyper-IL-6-treated BMDC-mediated Th17 polarization *in vitro*. **(A)** sgp130 suppresses IL-23 production by Hyper-IL-6-stimulated BMDCs. BMDCs were cultured in medium alone, with Hyper-IL-6 (100 nM), or with Hyper-IL-6 plus sgp130 (200 or 400 ng/mL) for 48 h. Culture supernatants were collected and IL-23 concentrations were quantified by ELISA. Data are presented as mean ± SEM (n = 5); ***P < 0.001, ****P < 0.0001 versus the Hyper-IL-6 group. **(B)** Representative flow cytometric plots showing the percentage of intracellular IL-17A expression in CD4^+^ T cells from (a) T cells, (b) DC+T cells, (c) Hyper-IL-6-DC+T cells, (d) Hyper-IL-6-DC+T cells/MSA, (e) Hyper-IL-6-DC+T cells/sgp130 (200 ng/mL) and (f) Hyper-IL-6-DC+T cells/sgp130 (400 ng/mL) group. **(C)** Quantitative analysis of the percentage of intracellular IL-17A expression in CD4^+^ T cells demonstrates sgp130 inhibits Th17 polarization induced by Hyper-IL-6-treated BMDCs. Data are presented as mean ± SEM (n = 5); *P < 0.05, ****P < 0.0001 versus the Hyper-IL-6-DC+T cells group. **(D)** ELISA analysis shows significant reductions in IL-23 level induced by Hyper-IL-6-treated BMDCs following sgp130 treatment. Data are presented as mean ± SEM (n = 5); ***P < 0.001, ****P < 0.0001 versus the Hyper-IL-6-DC+T cells group.

To specifically and potently activate the IL-6 *trans*-signaling pathway in a reductionist setting, we utilized Hyper-IL-6, a recombinant fusion protein of IL-6 and sIL-6R that directly and selectively engages gp130. This construct, while supra-physiological in its potency and bypassing natural sIL-6R shedding, serves as a precise tool to interrogate the sufficiency of *trans*-signaling activation in DCs. Emerging evidence indicates that Hyper-IL-6-stimulated BMDCs can potently drive Th17 differentiation via autocrine IL-23 production (34). To further define the contribution of IL-6 *trans*-signaling to BMDC-mediated Th17 polarization and to evaluate the effect of its selective blockade, we established an *in vitro* coculture system consisting of ovalbumin (OVA)-pulsed BMDCs and CD4^+^ T cells isolated from OVA-sensitized mice, in the presence or absence of Hyper-IL-6 and sgp130. Results showed that Hyper-IL-6 activation markedly increased IL-23 secretion by BMDCs (**Figure 6A**) and significantly elevated the frequency of IL-17A-producing CD4^+^ T cells in coculture (**Figures 6B, C**), consistent with IL-6/IL-23 axis-dependent Th17 induction. Notably, sgp130 treatment attenuated these responses, with high-dose sgp130 (400 ng/mL) significantly reducing IL-17A production and Th17 polarization (**Figures 6C, D**). Together, these results demonstrate that sgp130-mediated inhibition of IL-6 trans-signaling effectively limits BMDCs-driven Th17 responses *in vitro* while preserving the experimental context in which classical IL-6 signaling remains intact.

### sgp130 suppresses Th17 polarization mediated by Hyper-IL-6-activated BMDCs *in vivo*

**Figure 7:**
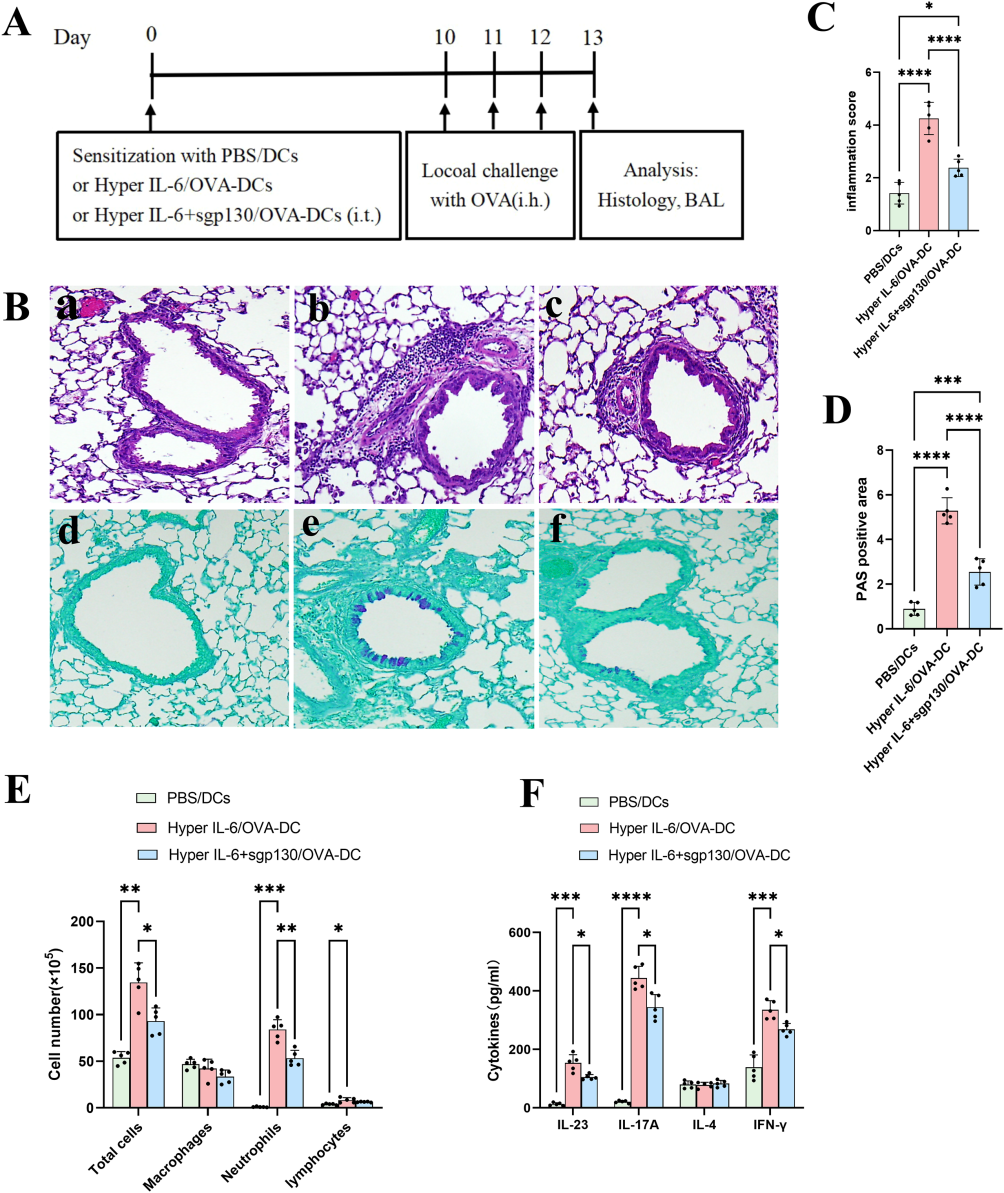
sgp130 attenuates Hyper-IL-6-treated BMDCs-mediated neutrophilic inflammation and Th17-associated responses *in vivo*. **(A)** Experimental design of the asthma model induced by adoptive transfer of BMDCs. Mice received intratracheal administration of: (i) PBS-treated, non-OVA-pulsed BMDCs (PBS/DCs, control); (ii) Hyper-IL-6-treated, OVA-pulsed BMDCs (Hyper-IL-6/OVA-DCs); or (iii) Hyper-IL-6 plus sgp130-treated, OVA-pulsed BMDCs (Hyper-IL-6+sgp130/OVA-DCs). Following DCs transfer (2 × 10^6^ cells/mouse), mice were challenged with OVA on days 10–12 and euthanized 24 h after the final challenge for analysis. **(B)** Representative histopathological analysis of lung tissues. Hematoxylin and eosin (H&E) staining (upper panels) illustrates decreased inflammatory cell infiltration in Hyper-IL-6+sgp130/OVA-DCs group mice compared with Hyper-IL-6/OVA-DCs group mice. Periodic acid–Schiff (PAS) staining (lower panels) shows weakened mucus production in Hyper-IL-6+sgp130/OVA-DCs group mice compared with Hyper-IL-6/OVA-DCs group mice. Images are shown for (a, d) PBS/DCs, (b, e) Hyper-IL-6/OVA-DCs, and (c, f) Hyper-IL-6+sgp130/OVA-DCs groups. **(C)** Semiquantitative scoring of peribronchial inflammation (scale 0–4) demonstrating significant improvement in Hyper-IL-6+sgp130/OVA-DCs group mice. Data are presented as mean ± SEM (n = 5); ****P < 0.0001 versus the Hyper-IL-6/OVA-DCs group mice. **(D)** Quantification of PAS-positive mucus-producing areas expressed as a percentage of the airway epithelial area, showing airway mucus hypersecretion decreases in Hyper-IL-6+sgp130/OVA-DCs group mice. Data are presented as mean ± SEM (n = 5); ****P < 0.0001 versus the Hyper-IL-6/OVA-DCs group mice. **(E)** Differential cell counts in BALF collected 24 h after the final OVA challenge. sgp130 treatment significantly reduced total cell numbers, with a pronounced decrease in neutrophils, compared with Hyper-IL-6/OVA-DCs group mice. Data are presented as mean ± SEM (n = 5); *P < 0.05, **P < 0.01, ***P < 0.001 versus the Hyper-IL-6/OVA-DCs group mice. **(F)** Cytokine concentrations in BALF collected 24 h after the final OVA challenge. ELISA analysis shows significant reductions in IL-23, IL-17A, and IFN-γ levels following sgp130 treatment, whereas IL-4 levels remain unchanged. Data are presented as mean ± SEM (n = 5); *P < 0.05, ***P < 0.001, ****P < 0.0001 versus the Hyper-IL-6/OVA-DCs group mice.

To determine whether sgp130 restrains Th17 polarization driven by Hyper-IL-6-activated BMDCs *in vivo*, we performed adoptive transfer experiments (**Figure 7A**). Histopathological examination revealed that pronounced peribronchial and perivascular inflammatory cell infiltration and mucus production in mice receiving Hyper-IL-6-treated, OVA-pulsed BMDCs, whereas these pathological features were markedly attenuated in mice receiving Hyper-IL-6 plus sgp130 treated, OVA-pulsed BMDCs (**Figures 7B–D**). In addition, mice receiving Hyper-IL-6-treated, OVA-pulsed BMDCs developed pronounced neutrophilic airway inflammation, as evidenced by increased neutrophil numbers in BALF (**Figure 7E**), together with an enhanced Th17/Th1-associated cytokine profile, including elevated IL-23, IL-17A, and IFN-γ levels in BALF (**Figure 7F**). In contrast, transfer of BMDCs treated with sgp130 in addition to Hyper-IL-6 significantly attenuated BALF neutrophilia (**Figure 7E**) and reduced Th17-associated cytokine production, as indicated by decreased IL-23 and IL-17A concentrations (**Figure 7F**). Collectively, these results demonstrate that sgp130 limits the capacity of Hyper-IL-6-activated BMDCs to elicit neutrophilic inflammation and Th17-skewed immune responses *in vivo*.

## Discussion

Th17 cells are key drivers of neutrophilic asthma, an asthma endotype defined by predominant airway neutrophilia and closely linked to Th17-skewed immune responses (35). The Th17-neutrophil axis is particularly evident in severe asthma, in which elevated IL-17A levels in bronchoalveolar lavage fluid are associated with airway remodeling and glucocorticoid resistance (36, 37). Emerging evidence supports a central role for the Th17-IL-17A axis in neutrophilic airway inflammation through multiple, convergent mechanisms. At the signaling level, IL-17 receptor engagement enhances potent cellular responses that impact diverse diseases (38). Beyond these cell-intrinsic pathways, innate-adaptive immune crosstalk further amplifies inflammatory cascades. In particular, the IL-17C/IL-17RE axis has been proposed as a molecular switch that promotes pro-inflammatory macrophage (M1) polarization, potentially establishing a feed-forward loop that sustains neutrophilic inflammation (39). Within immunopathological profile of neutrophilic asthma, the IL-23-Th17 axis also represents a central regulatory pathway. IL-23 promotes Th17 expansion and persistence and can amplify neutrophilic inflammation through interconnected cytokine networks (40, 41). Collectively, these observations support the IL-23-Th17-neutrophil axis as a potential therapeutic target in neutrophilic asthma.

The IL-6/sIL-6R complex is a key mediator of IL-6 trans-signaling, a pathway implicated in the pathogenesis of neutrophilic asthma (16, 42). This severe asthma phenotype, often characterized by predominant airway neutrophilia (e.g., ≥71% sputum neutrophils) with relative eosinophil paucity, remains challenging to treat because of its marked corticosteroid resistance (43). Notably, an IL-6/sIL-6R-responsive gene signature is enriched in non-eosinophilic asthma endotypes and inversely correlates with eosinophil counts in clinical samples (16, 44). This pattern likely reflects differential engagement of IL-6 signaling modalities, namely classical IL-6 signaling via membrane-bound IL-6R is confined to selected cell types (e.g., lymphocytes and monocytes), whereas sIL-6R-dependent *trans*-signaling can act broadly on gp130-expressing cells such as AECs and fibroblasts (45, 46). In airway epithelial cells (AECs), IL-6 trans-signaling induce rapid and sustained JAK2-dependent phosphorylation of STAT3 at Tyr705, thereby upregulating pro-inflammatory programs linked to neutrophil recruitment and chronic inflammation (16, 47). This response includes increased expression of chemokines (e.g., CXCL8/IL-8) and adhesion molecules through STAT3-dependent transcriptional activation (48, 49). In addition, bronchial fibroblasts exposed to IL-6 *trans*-signaling display enhanced production of MCP-1 (CCL2), which may further reinforce inflammatory cascades (16). Together, these observations support IL-6 *trans*-signaling as a candidate biomarker for asthma endotyping, particularly in steroid-refractory disease associated with Th17-skewed inflammation. Therapeutically, targeting this axis may allow preferential inhibition of pathogenic *trans*-signaling while preserving homeostatic functions mediated by classical IL-6 signaling.

In this study, we extend these observations by providing evidence that IL-6 *trans*-signaling in pulmonary antigen-presenting cells contributes to Th17-associated neutrophilic inflammation, and that local sgp130 administration attenuates this pathogenic axis. Consistent with prior reports linking Th17 to neutrophilic airway inflammation, we observed increased IL-17A levels in BALF in our murine model. Importantly, sgp130, a selective inhibitor of IL-6 *trans*-signaling, reduced Th17 polarization and decreased IL-23 expression in pulmonary CD11c^+^ antigen-presenting cells (APCs), supporting a functional connection between IL-6 *trans*-signaling and the IL-23/IL-17 pathway in this setting. We propose IL-6 *trans*-signaling in the lung potentially acting on multiple cell types including CD11c^+^ APCs, fosters a cytokine environment characterized by elevated IL-23. This IL-23-rich milieu is strongly linked to the expansion and stabilization of pathogenic Th17 cells, driving neutrophilic inflammation. While our study position IL-23 as a key downstream mediator, formal proof of its absolute requirement within this specific pathway would require IL-23 blockade or genetic deletion experiments in conjunction with our model. In parallel, we detected reduced membrane-bound IL-6 receptor (mIL-6R) expression on pulmonary DCs from asthmatic mice. Accumulating evidence indicates that sIL-6R is generated predominantly through proteolytic cleavage of mIL-6R. In inflammatory settings, including fracture healing and arthritis, ADAM17-mediated ectodomain shedding is a major route for sIL-6R production and thereby promotes IL-6 trans-signaling with pro-inflammatory consequences (50, 51). We speculate enhanced mIL-6R shedding and a relative shift from classical IL-6 signaling toward IL-6 *trans*-signaling in our study, thereby amplifying pro-inflammatory IL-6 activity within the airway microenvironment and contributing to persistent inflammation. However, we acknowledge the possibility of indirect effects of sgp130 on classical signaling. For instance, by reducing the availability of IL-6 in the microenvironment, sgp130 may secondarily dampen signaling through the membrane-bound receptor. Our data do not delineate these potential secondary effects. Future experiments using cell-specific deletion of membrane IL-6Rα (to ablate classical signaling) in conjunction with sgp130 treatment would be required to definitively partition the contributions of each pathway to the observed anti-inflammatory phenotype.

To better isolate the contribution of dendritic cell (DC)-associated IL-6 trans-signaling from systemic effects on other effector cells, we performed adoptive transfer experiments in which Hyper-IL-6-activated DCs were pretreated *in vitro* with soluble gp130 (sgp130) before administration to naïve recipients. Transfer of Hyper-IL-6-activated DCs induced neutrophilic airway inflammation accompanied by increased Th17-associated cytokines, including IL-23 and IL-17A, consistent with the established capacity of activated DCs to promote Th17 polarization through IL-6- and IL-23-dependent mechanisms (52). The use of Hyper-IL-6 in our reductionist experiments provides clear evidence for the sufficiency of IL-6 trans-signaling activation in driving DC-mediated Th17 responses. We interpret these findings as a validated mechanistic proof-of-concept. The physiological scenario likely involves more modulated signaling via the dynamically regulated pool of endogenous IL-6 and sIL-6R, but our data confirm that this pathway is capable of steering immunity towards a Th17 outcome when engaged. Notably, sgp130 pretreatment of DCs before transfer reduced BALF neutrophilia and decreased IL-23 and IL-17A levels, supporting the interpretation that IL-6 *trans*-signaling within transferred DCs contributes to Th17-skewed airway inflammation. It is important to emphasize that the therapeutic effects of intratracheal sgp130 are almost certainly pleiotropic, impacting IL-6 *trans*-signaling in alveolar epithelial cells, macrophages, and other lung resident cells. Future studies employing cell-specific gp130 knockout models would be required to precisely partition the contributions of different lung cell populations. In addition to modulating the IL-23/Th17 axis, the potential impact of sgp130 on additional cytokine pathways, including granulocyte-macrophage colony-stimulating factor (GM-CSF), is acknowledged as an important area for future investigation to fully delineate the mechanism of action. Together, these findings reinforce a model in which DC-associated IL-6 *trans*-signaling promotes Th17 polarization and neutrophilic inflammation and suggest that sgp130-based blockade may be a rational strategy to modulate this pathogenic pathway in asthma.

Several limitations are noted in this study. First, a methodological consideration in this study is the use of positive selection kits (CD11c^+^ microbeads) for the isolation of lung dendritic cells. While this method yields cells of high purity, it is recognized that antibody binding during positive selection could theoretically alter cell surface receptor density or induce subtle activation signals. Although our *in vitro* stimulation assays were conducted after an overnight rest period to minimize acute isolation effects, we cannot fully rule out that the isolation method may have influenced the basal state of the cells. Future studies employing negative selection or fluorescence-activated cell sorting (FACS) could corroborate our findings. Second, our findings are derived from an OVA+LPS murine model, which elicits a neutrophilic airway infiltrate and a pronounced Th17 response, mirroring some key features of human neutrophilic asthma. This model is pertinent as it demonstrates elevated IL-6 levels and IL-6-dependent pathology, similar to subsets of severe asthma characterized by increased airway IL-6 and IL-17. However, we acknowledge the limitations of this acute model in fully capturing the chronicity, heterogeneity, and remodelling aspects of human disease. Nevertheless, the model provides a validated platform to dissect the IL-6/IL-23/IL-17 axis, which is increasingly implicated in neutrophilic inflammation across species. Our results position IL-6 *trans*-signaling as a potential target within this pathway, a hypothesis that now requires testing in more chronic models and ultimately in clinical cohorts stratified by IL-6 and neutrophilic biomarkers.

In summary, our data show that local administration of soluble gp130 (sgp130) attenuates key features of Th17-associated neutrophilic airway inflammation, consistent with inhibition of IL-6 *trans*-signaling in the pulmonary compartment. These findings support a pathogenic role for IL-6 *trans*-signaling in neutrophilic asthma and suggest that targeting this pathway can modulate Th17 polarization and downstream inflammatory readouts. Clinically, it will be important to determine whether markers of IL-6 *trans*-signaling can aid asthma endotyping and predict disease severity, particularly in corticosteroid-refractory phenotypes. Future studies should further define the cell-type-specific mechanisms of sgp130 *in vivo* and evaluate the possibility and translational relevance of *trans*-signaling blockade in asthmatic patients with airway neutrophilic inflammation and steroid resistance. Together, our work may inform IL-6/sIL-6R complex as a valuable molecular biomarker in neutrophilic asthma, and blocking IL-6 *trans*-signaling may be a precision strategie for therapy.

## Data Availability

The raw data supporting the conclusions of this article will be made available by the authors, without undue reservation.

## Supplementary material

Supplementary Figure 1 Schematic overview of the experimental strategy for detecting pulmonary Th17 cells.

Supplementary Figure 2 Schematic overview of the experimental strategy for pulmonary Treg detection.

Supplementary Figure 3 Schematic overview of the experimental strategy for assessing IL-6R expression on CD11c^+^ APCs in lung tissue.

Supplementary Figure 4 Schematic overview of the experimental strategy used to assess IL-23 expression in pulmonary CD11c^+^ APCs.
