# Supplementary figures and images for: Soluble gp130 inhibits Th17 polarization in neutrophilic asthma by blocking IL-6 trans-signaling in dendritic cells
Source: Front Immunol. 2026 Mar 27;17:1787115. doi: 10.3389/fimmu.2026.1787115 (PMC13065515; doi:10.3389/fimmu.2026.1787115)

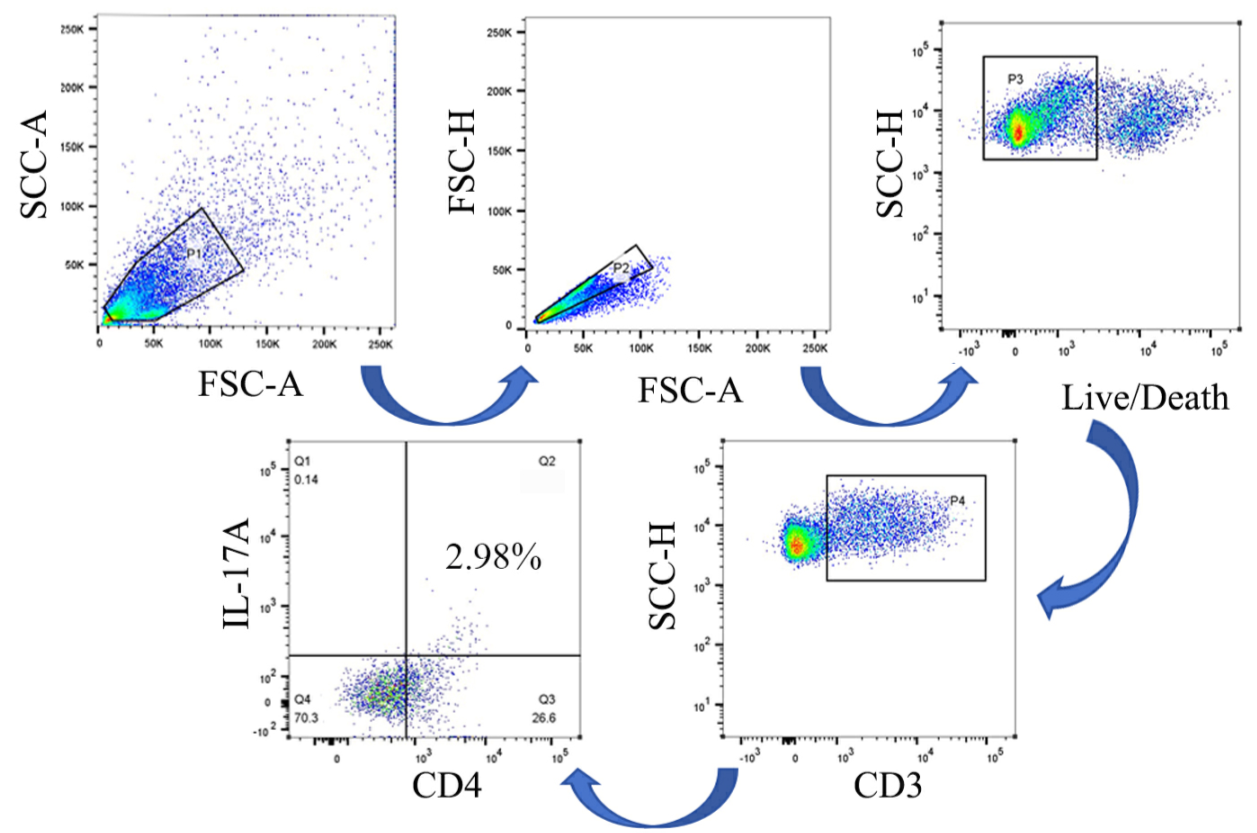

Supplement: Supplementary Figure 1 — Schematic overview of the experimental strategy for detecting pulmonary Th17 cells. [file Image1.tif]

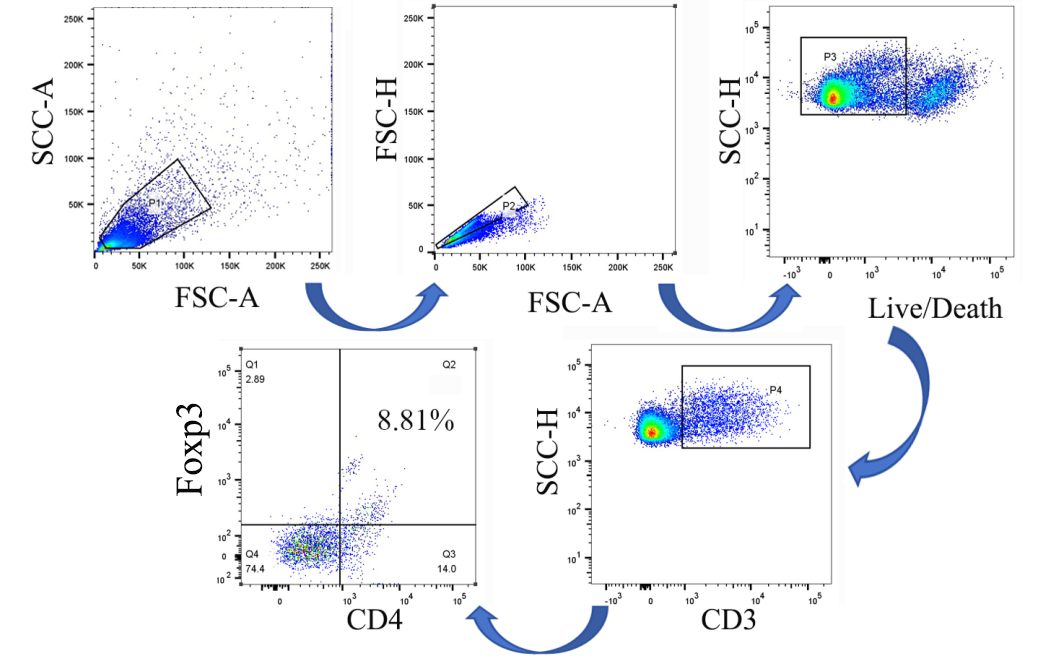

Supplement: Supplementary Figure 2 — Schematic overview of the experimental strategy for pulmonary Treg detection. [file Image2.tif]

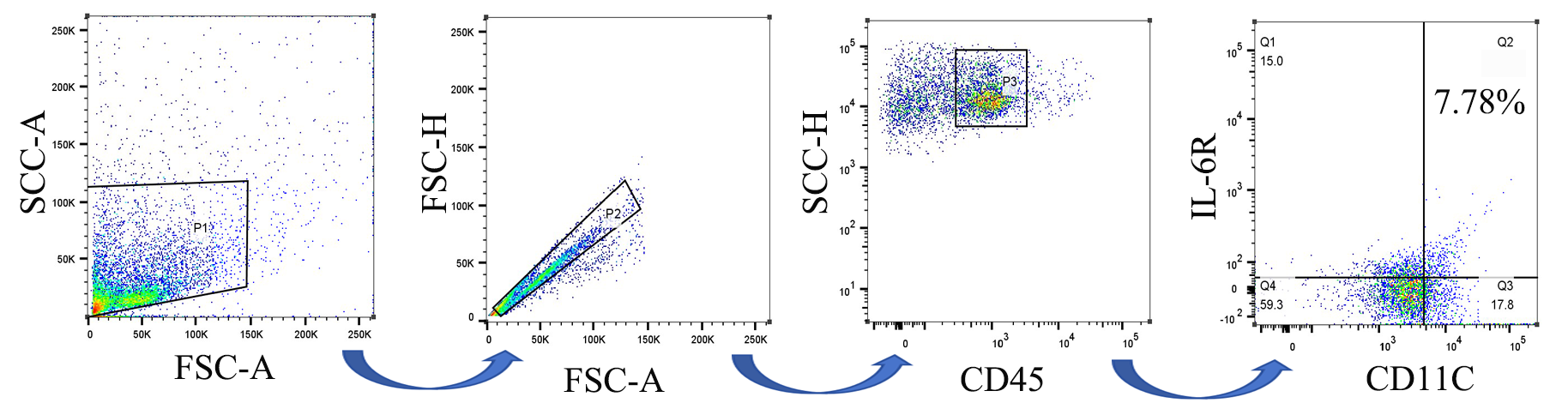

Supplement: Supplementary Figure 3 — Schematic overview of the experimental strategy for assessing IL-6R expression on CD11c+ APCs in lung tissue. [file Image3.tif]

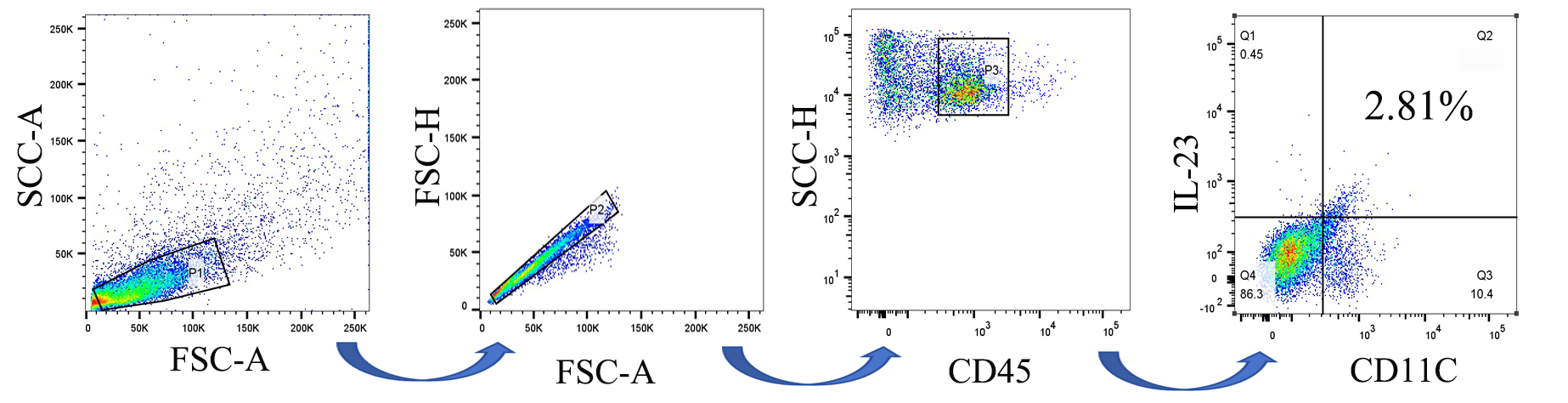

Supplement: Supplementary Figure 4 — Schematic overview of the experimental strategy used to assess IL-23 expression in pulmonary CD11c+ APCs. [file Image4.tif]
